# A Single-Center Retrospective Analysis of a Standardized Sedation Protocol for MRI in Children with Achondroplasia: Minimal Complications and Excellent Imaging Quality

**DOI:** 10.3390/children12060662

**Published:** 2025-05-22

**Authors:** Barbora Nedomová, Lucia Babulicová, Ľubica Tichá, Salome Jakešová, Ladislava Wsólová, Rudolf Riedel

**Affiliations:** 1 Department of Pediatric Anesthesiology and Intensive Medicine, Faculty of Medicine, Comenius University and National Institute of Children’s Diseases, 83340 Bratislava, Slovakia; lucia.babulicova@nudch.eu (L.B.); rudolf.riedel@nudch.eu (R.R.); 2 Faculty of Medicine, Slovak Medical University, 83101 Bratislava, Slovakia; 3 Department of Pediatrics, Faculty of Medicine, Comenius University and National Institute of Children’s Diseases, 83340 Bratislava, Slovakia; lubica.ticha@nudch.eu; 4 Department of Radiology, National Institute of Children’s Diseases, 83340 Bratislava, Slovakia; salome.jakesova@nudch.eu; 5 Institute of Biophysics, Informatics and BioStatistics, Faculty of Public Health, Slovak Medical University, 83101 Bratislava, Slovakia; ladislava.wsolova@szu.sk

**Keywords:** achondroplasia, children, sedation, midazolam, propofol

## Abstract

**Background/Objectives:** Achondroplasia, the most common form of skeletal dysplasia, poses significant challenges for procedural sedation due to distinct anatomical and physiological features. This study evaluated the safety, effectiveness, and imaging quality of a standardized sedation protocol for magnetic resonance imaging (MRI) in children with achondroplasia. **Methods:** We conducted a single-center, retrospective analysis of 22 MRI procedures in 12 pediatric patients. Intravenous midazolam and propofol were used as primary sedatives, with continuous monitoring and standardized dosing. Sedation parameters were compared between children aged <1 year and children aged ≥1 year. **Results:** The median sedation duration was 35 (25–65) min, and the median recovery time was 9 (5–14) min. No significant differences were found between age groups in midazolam dose (<1 year: 0.15 ± 0.05 mg/kg vs. ≥1 year: 0.13 ± 0.04 mg/kg; *p* = 0.238), propofol induction (2.26 ± 1.14 vs. 1.80 ± 0.52 mg/kg; *p* = 0.375), or infusion rate (3.18 ± 2.74 vs. 5.13 ± 2.65 mg/kg/h; *p* = 0.203), indicating protocol consistency. High-quality images were obtained in all cases. In one case (4.5%), self-limited desaturation to 92% occurred, with no intervention required. No airway instrumentation or other complications were reported. **Conclusions:** This protocol provides safe, effective, and reproducible sedation in children with achondroplasia, ensuring high-quality MRI with minimal adverse events.

## 1. Introduction

Achondroplasia is a rare genetic disorder with an incidence of approximately one per 20,000–30,000 live births [1,2]. It is caused by a mutation in the fibroblast growth factor receptor 3 gene, leading to abnormal endochondral ossification and characteristic skeletal dysplasia [3]. Key challenges in procedural sedation may include foramen magnum stenosis (FMS), craniofacial features associated with difficult airway management, obstructive sleep apnea (OSA), impaired ventilation due to reduced respiratory reserve, and difficult intravenous access (DIVA), depending on individual patient characteristics [4].

FMS is the most severe and potentially fatal complication in infancy and early childhood, with an estimated mortality rate of 2–7.5% [5,6,7]. It may lead to brainstem compression, central apnea, respiratory insufficiency, and sudden death [8,9]. Given this risk, a thorough neurological assessment and polysomnography to evaluate sleep-related breathing disorders are essential before procedural sedation. Excessive neck extension or head manipulation during airway management can exacerbate brainstem compression, highlighting the critical importance of meticulous patient positioning to prevent such complications.

Airway characteristics—including midface hypoplasia, depressed nasal bridge, relatively large mandible, small mouth opening, narrow nostrils, macroglossia, adeno-tonsillar hypertrophy, nasal and laryngeal hypoplasia, tracheobronchomalacia, and a short, rigid neck with limited mobility—significantly complicate mask ventilation, laryngoscopy, and intubation. OSA, which is frequently associated with midface hypoplasia and macroglossia, further exacerbates the risk of upper airway obstruction during sedation.

Additionally, reduced spinal mobility and anatomical variations in vertebral structures complicate patient positioning, adding complexity to procedural sedation management. Ventilatory impairment, resulting from reduced thoracic volume and limited respiratory reserve, further increases respiratory risks during sedation.

Given these anatomical and physiological considerations, procedural sedation in children with achondroplasia requires a precise, risk-adapted approach to airway management, ventilation, intravenous access, and patient positioning. MRI is essential for the early detection of cervicomedullary compression and is crucial in guiding clinical management [5,6]. However, procedural sedation for MRI in children with achondroplasia presents substantial challenges. The optimal sedation depth is fundamental for obtaining high-quality imaging. Insufficient sedation can cause motion artifacts and non-diagnostic images, whereas excessive sedation significantly increases the risk of respiratory complications. Routine pediatric sedation principles alone are insufficient for this high-risk population, and standardized guidelines for safe procedural sedation in children with achondroplasia undergoing MRI are currently lacking.

In this study, we systematically assessed the respiratory and cardiovascular complications associated with procedural sedation for MRI in children with achondroplasia. Additionally, we aimed to review the current knowledge, emphasizing the need for further research and the establishment of clear, evidence-based sedation protocols.

## 2. Materials and Methods

A retrospective, single-center observational study was conducted at the National Institute of Children’s Diseases in Bratislava, Slovakia. This study included pediatric patients with a confirmed diagnosis of achondroplasia who underwent magnetic resonance imaging (MRI) between 1 January 2010 and 31 December 2023. The study protocol was approved by the institutional ethics committee (Approval ID: EK 2/2/24), and written informed consent was obtained from the parents or legal guardians of all participants.

The primary aim of this study was to evaluate the safety of a standardized procedural sedation approach and the diagnostic yield of MRI in pediatric patients with achondroplasia. We analyzed demographic characteristics, types of sedation used, administered sedative doses, sedation duration, recovery time, complications related to sedation, and MRI completion rates. We also compared sedation parameters between children under and over the age of one year. Additional data collected included the American Society of Anesthesiologists (ASA) physical status classification.

### 2.1. Patient Selection and Pre-Sedation Evaluation

A retrospective review was conducted on pediatric patients with a genetically confirmed diagnosis of achondroplasia who underwent MRI examinations under procedural sedation using a standardized protocol. The study population comprised 12 children (6 males and 6 females), aged between 3 weeks and 5 years, who required sedation to complete the MRI scan.

The inclusion criteria were as follows: (1) confirmed diagnosis of achondroplasia and (2) need for procedural sedation to complete the MRI. The exclusion criteria included the following: (1) lack of informed consent; (2) known contraindications to midazolam or propofol; (3) acute illness at the time of imaging (e.g., upper respiratory tract infection, fever, vomiting, or clinical instability); and (4) use of an alternative sedation method outside the standardized protocol (e.g., chloral hydrate).

All patients underwent neurological and respiratory assessments prior to the procedure, including polysomnography, where indicated, to evaluate risk factors for snoring and obstructive sleep apnea.

A pediatric anesthesia consultant conducted a comprehensive pre-sedation evaluation, which included fasting status, detailed medical history, current medications, allergies, previous sedation experiences, and prior adverse reactions to anesthesia. Particular attention was given to evaluating risks related to airway obstruction, obstructive sleep apnea, altered respiratory mechanics, and potential spinal cord compression.

### 2.2. Sedation Protocol

All patients received a standardized intravenous sedation protocol consisting of midazolam and propofol. An intravenous cannula was placed prior to sedative administration; in one patient with DIVA, near-infrared (NIR) imaging was used to facilitate cannulation.

The protocol included the following steps:

- Premedication: Midazolam (Accord Healthcare GmbH, Munich, Germany), 0.1–0.2 mg/kg IV, administered before transfer to the MRI holding area.
- Induction: 1% propofol (B. Braun Melsungen AG, Melsungen, Germany), 1–2 mg/kg IV bolus.
- Maintenance: Continuous infusion of 1% propofol at 4 mg/kg/h, titrated by 0.5–1 mg/kg/h as needed based on clinical response and target sedation depth.

The protocol was designed to achieve deep sedation while minimizing potential adverse effects such as bradycardia, hypotension, and oxygen desaturation.

The target sedation depth was defined as level 3 on the University of Michigan Sedation Scale (UMSS) [10], corresponding to deep sedation with maintained spontaneous ventilation, stable oxygenation, and no need for airway support. The UMSS ranges from 0 (awake) to 4 (unresponsive to deep stimulation) and was used throughout the procedure to monitor and document sedation depth. Sedation deeper than UMSS 3 was intentionally avoided due to the elevated risk of airway obstruction and hypoventilation in children with achondroplasia.

If sedation was deemed insufficient—based on a UMSS score < 3, excessive movement, or discomfort compromising image quality—an additional bolus of propofol (0.5–1 mg/kg IV) was administered.

The protocol also included predefined safety interruption criteria. If any of the following signs occurred, sedation was paused, and the patient was temporarily removed from the MRI scanner:

- signs of airway obstruction (e.g., paradoxical breathing, persistent coughing);
- oxygen desaturation (SpO_2_ < 90%);
- hypercapnia (end-tidal carbon dioxide [EtCO_2_] > 6.5 kPa);
- bradycardia (>20% decrease in heart rate from baseline);
- or any clinically significant arrhythmia.

In such cases, the anesthesiology team promptly assessed and stabilized the patient before resuming or modifying the sedation regimen.

### 2.3. Periprocedural Management

#### 2.3.1. Airway Management and Ventilation

Airway patency was maintained through neutral head and neck positioning, avoiding cervical extension. Supplemental oxygen (2–3 L/min) was administered via a pediatric face mask. SpO_2_, EtCO_2_, and respiratory rate (RR) were continuously monitored to ensure adequate ventilation.

#### 2.3.2. Monitoring System

A camera system in the MRI suite provided continuous visual surveillance of the patient throughout the procedure. An MRI-compatible vital signs monitor was used to record physiological parameters. The anesthesiologist continuously monitored and recorded the heart rate (HR), SpO_2_, RR, and EtCO_2_ at 5-min intervals. Sedation depth was assessed using the UMSS at baseline, during sedation, and throughout recovery. Non-invasive arterial blood pressure was measured before sedative administration, immediately prior to MRI, and after completion. Hypotension was defined as a >20% decrease in mean arterial pressure from baseline. All data were recorded manually by the anesthesiologist using paper-based anesthesia records, in accordance with ASA monitoring standards.

#### 2.3.3. Recovery and Discharge

Propofol infusion was discontinued immediately upon completion of the MRI. Patients were transferred to the recovery area, where an anesthesia nurse monitored heart rate and SpO_2_ until discharge criteria were met. Readiness for discharge was defined as a modified Aldrete score > 9, based on activity, respiration, circulation, consciousness, and oxygen saturation. Once stable, patients were returned to the pediatric daycare ward under medical and nursing supervision prior to discharge home.

### 2.4. Statistical Analysis

Data were statistically analyzed using SPSS version 28 (IBM Corp., Armonk, NY, USA). All anesthetic doses are expressed as mean ± standard deviation. Anesthetic doses administered to pediatric patients aged <1 and ≥1 year were compared using independent two-tailed *t*-tests or non-parametric independent two-tailed Mann–Whitney tests. Values were considered significantly different at α = 0.05.

## 3. Results

Between January 2010 and December 2023, 30 MRI procedures were performed in pediatric patients with achondroplasia at our institution. Of these, eight procedures were excluded from the analysis: seven because sedation was not required and one owing to the use of chloral hydrate instead of the standardized sedation protocol.

In the seven excluded procedures without sedation, the children were calm and cooperative, and the examinations could be completed without movement, resulting in diagnostic-quality images without the need for pharmacological support.

Chloral hydrate was used in one patient based on a history of successful previous sedation with this agent and at the parents’ explicit request. The anesthesiology team agreed to this decision. As this method did not follow the standardized protocol under investigation, the case was excluded from analysis.

**Figure 1:**
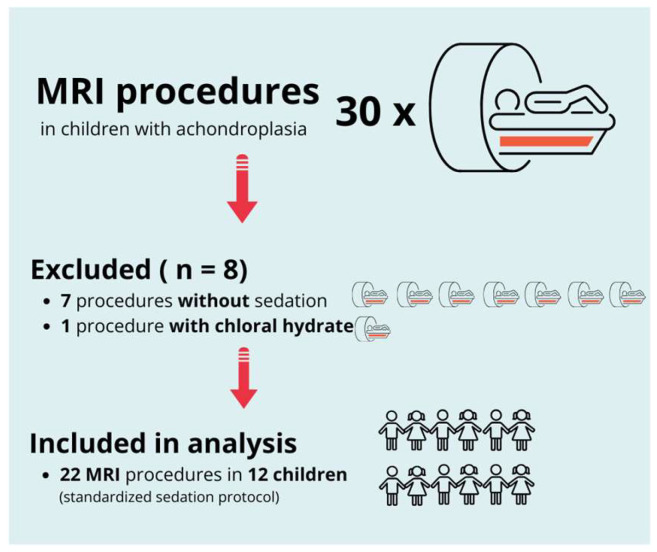
Flow diagram of patient and procedure selection. From an initial total of 30 MRI procedures in children with achondroplasia, 8 were excluded (7 without sedation and 1 with chloral hydrate). The final analysis included 22 MRI procedures in 12 children, all performed under a standardized sedation protocol.

The final analysis included 22 MRI procedures in 12 pediatric patients, all of whom received sedation using the standardized protocol consisting of midazolam and propofol. All procedures were conducted to evaluate brain or spinal abnormalities associated with achondroplasia. Figure 1 presents a flow diagram outlining the selection process. Table 1 summarizes patient demographics and the number of sedated MRI procedures per patient.

All 22 MRI procedures were conducted under procedural sedation using a standardized protocol and multimodal monitoring. All patients received midazolam (0.14 ± 0.044 mg/kg) before propofol induction. Propofol was administered with an induction dose of 1.93 ± 0.739 mg/kg, followed by a continuous infusion of 4.60 ± 2.76 mg/kg/h. The total propofol dose was 4.83 ± 1.99 mg/kg.

The median duration of procedural sedation was 35 (25, 65) min. Recovery time was defined as the interval between cessation of sedative administration and fulfillment of all recovery room discharge criteria: return to baseline consciousness, stable vital signs, effective spontaneous ventilation, and SpO_2_ > 94% on room air. The median recovery time was 9 (5, 14) min (Table 2).

**Figure 2:**
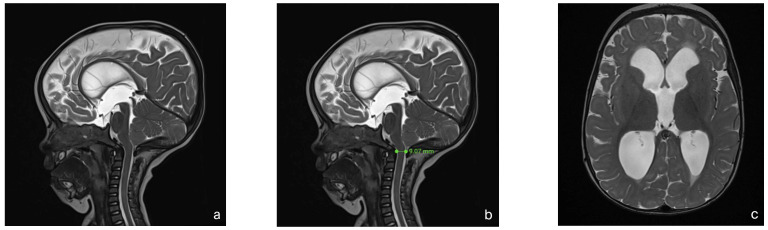

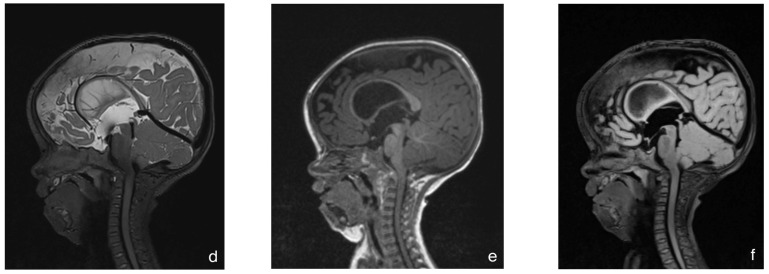
MRI of an 8-month-old child with achondroplasia. (**a**) Sagittal T2-weighted magnetic resonance image (MRI) shows foramen magnum stenosis with normal signal in the cervical spinal cord. (**b**) Sagittal T2-weighted MRI highlights a reduced dens–opisthion distance, with the anteroposterior diameter at the C2–C0 level measuring 9 mm. (**c**) Axial T2-weighted MRI shows ventriculomegaly. (**d**) Sagittal T2-weighted SPACE image demonstrates reduced perimedullary cerebrospinal fluid (CSF) flow void signal at the cervicocranial junction. (**e**) Sagittal T1-weighted image shows no evidence of cerebellar tonsillar herniation. (**f**) Sagittal T2-weighted FLAIR image shows no signs of cervical myelopathy.

With this standardized procedural sedation approach and continuous multimodal monitoring, none of the patients required endotracheal intubation or a laryngeal mask airway. All MRI scans were reviewed by a board-certified pediatric radiologist and deemed of sufficient quality for diagnostic purposes. No repeat imaging was required due to motion artifacts. Figure 2 presents a representative MRI image acquired under the standardized sedation protocol.

The mean midazolam dose did not differ significantly between children aged <1 year (0.15 ± 0.05 mg/kg) and those aged ≥1 year (0.13 ± 0.04 mg/kg; *p* = 0.238). Similarly, propofol doses for induction (2.26 ± 1.14 mg/kg vs. 1.80 ± 0.52 mg/kg; *p* = 0.375) and infusion rates (3.18 ± 2.74 mg/kg/h vs. 5.13 ± 2.65 mg/kg/h; *p* = 0.203) showed no significant differences between the two age groups. Procedural sedation with midazolam combined with propofol enabled high-quality imaging with minimal adverse effects. Only one transient respiratory event was observed—a self-limiting desaturation episode (nadir SpO_2_ of 92%) that resolved spontaneously without the need to interrupt the imaging procedure. One case of DIVA occurred. No postprocedural respiratory or cardiovascular complications were recorded in any patient.

## 4. Discussion

Information regarding anesthesia or procedural sedation specifically for MRI in children with achondroplasia is limited. To our knowledge, this study represents the first systematic retrospective evaluation of sedation-related complications during MRI procedures in pediatric patients with achondroplasia. Our findings demonstrate that a standardized procedural sedation protocol using midazolam and propofol provides effective and safe sedation without the need for airway instrumentation. No major complications were observed, and diagnostic image quality was preserved in all cases.

MRI is crucial in screening for cervicomedullary compression in children with achondroplasia, with recommendations evolving over the years. Although earlier guidelines from the American Academy of Pediatrics advocated routine screening with MRI or CT in neonates [11,12], more recent consensus recommendations favor a selective approach based on clinical findings or polysomnography [13]. However, this selective strategy remains controversial, as some studies have highlighted the risk of overlooking clinically significant spinal cord compression in the absence of imaging examinations [14,15]. The 2022 International Consensus strongly recommends routine MRI screening for FMS during the first months of life [3]. Recent European guidelines have gone even further, emphatically advocating for MRI between 3 and 6 months of age [5]. Given that MRI in this age group almost invariably requires sedation or anesthesia, which carries potential serious risks, it is critical for anesthesiologists to strictly adhere to the standardized safety protocols. Our findings conclusively prove that implementing a precise sedation protocol with individualized dosing and continuous monitoring significantly enhances patient safety while ensuring high-quality diagnostic imaging. This fundamentally contributes to early risk stratification and effective intervention planning.

The administration of procedural sedation or anesthesia in children with achondroplasia presents unique and multifactorial challenges, including difficult airway management, central and obstructive sleep apnea, specific positioning requirements, and difficulties with vascular access. These challenges necessitate meticulous preparation and close interdisciplinary collaboration.

Given the anatomical and functional characteristics of this population, airway management planning must be based on thorough preprocedural anesthetic evaluation. Although sedation is usually sufficient, the anesthesia team must remain prepared for potential escalation to general anesthesia and airway instrumentation if complications arise. Appropriate equipment and backup strategies must be readily available to manage difficult airway scenarios in these patients.

Abnormal bone growth in achondroplasia significantly complicates mask ventilation due to atypical craniofacial anatomy. Neck extension should be minimized or completely avoided, even in the absence of a confirmed diagnosis of foramen magnum stenosis, given its high prevalence and the potential risk of cervicomedullary compression. These precautions, however, may limit glottic visualization if intubation becomes necessary. A head ring is recommended to optimize airway management conditions and stabilize the cervical spine. Video laryngoscopy should always be readily available and is considered the preferred technique in these cases [16].

Earlier studies suggested that patient weight may be a more appropriate parameter for selecting endotracheal tube size, as it correlates more directly with laryngeal size [17,18]. However, recent evidence supports the traditional approach of using patient age, which correlates with head and laryngeal development [19]. When supraglottic airway devices such as laryngeal masks are used, anatomical features—including a small oral cavity and macroglossia—may necessitate the selection of a smaller size than typically expected.

In a study by Monedero et al., intubation-related complications occurred in only one out of 15 pediatric patients who underwent a total of 53 orthopedic procedures. The authors attributed this finding to the relatively older age of the cohort (all children were >4 years old) [17].

The risk of upper airway obstruction and cervical cord compression is highest during the first year of life [17], making it essential to anticipate potential airway difficulties in this population [20]. Comprehensive anatomical understanding, careful selection of airway devices, and gentle cervical spine manipulation—including the avoidance of hyperextension—are critical for safe anesthetic management in these children.

Given these airway and anatomical complexities, selecting the most appropriate induction technique is essential. Intravenous induction is preferred when feasible, as securing venous access prior to airway manipulation allows for immediate administration of emergency medications and ensures greater control in the event of airway complications. However, intravenous access may be challenging due to excessive skin laxity, soft tissue bulk, and joint contractures [19,21]. In our cohort, only one child met the criteria for DIVA, which was successfully managed using NIR imaging. NIR is effective for visualizing superficial subcutaneous veins, typically located at depths of up to 6–7 mm. For deeper or poorly visualized veins, ultrasound guidance is preferred and represents the standard of care in such cases.

Optimal sedation strategies are equally pertinent. In our experience, the combination of propofol and midazolam, administered by a pediatric anesthesiologist, provided sufficient sedation depth and immobility and was associated with a low incidence of respiratory events and good tolerability across all pediatric age groups. No significant differences in dosing were observed between infants under 1 year of age and older children.

Adverse events were minimal, with only one mild respiratory incident reported. Importantly, all MRI scans were of high diagnostic quality, and no repeat imaging was necessary. These findings support the feasibility and safety of a standardized sedation protocol for MRI in children with achondroplasia. By ensuring both patient safety and diagnostic accuracy, this approach facilitates the early detection of foramen magnum stenosis and timely intervention planning in accordance with current international guidelines.

Pediatric procedural sedation during MRI can be achieved using a range of intravenous sedatives, such as propofol, midazolam, ketamine, or dexmedetomidine [22]. However, propofol as a sole agent is associated with an increased incidence of serious sedation-associated adverse events owing to a dose-dependent response to upper airway collapse by inhibiting airway dilator muscles and upper airway reflexes. Therefore, combinations of propofol with other sedatives, such as midazolam, ketamine, or dexmedetomidine, have been evaluated for pediatric procedural sedation. Propofol and midazolam are preferred over other intravenous sedatives for pediatric procedural sedation because of their high potency, short half-life, and low potential for adverse effects [23]. When determining sedation dosing strategies for children, clinicians must consider the child’s clinical condition, type of procedure or examination, and the anticipated pain levels.

Propofol is widely used for pediatric sedation because of its rapid onset and quick recovery profile. However, guidelines regarding appropriate propofol dosing in children undergoing radiological examinations remain unclear. Current recommendations suggest a range of propofol doses based on body weight [19]. In our study, anesthesiologists adhered to a similar weight-based dosing approach, selecting propofol doses within the recommended ranges according to each child’s clinical condition, individual anesthesiologist experience, and preference [24]. We employed a conservative, evidence-based propofol dosing regimen of 3–5 mg/kg/h, reflecting a more conservative and safety-oriented approach to pediatric MRI sedation, similar to the experience described by Johnson et al., who retrospectively analyzed the use of lower propofol doses in pediatric practice [25].

Although our study offers preliminary guidance for procedural sedation in pediatric patients with achondroplasia, further research is needed. Larger, multicenter studies are required to validate our findings. Further research into the genetic and physiological factors influencing sedation responses in these patients may lead to the development of individualized sedation protocols, thereby significantly enhancing patient safety and MRI quality. Based on our findings and the current body of evidence, we propose a set of practical recommendations for procedural sedation during MRI in children with achondroplasia, as summarized in Table 3.

Our study has certain limitations. The retrospective design and relatively small sample size may limit the generalizability of our findings to the broader population of pediatric patients with achondroplasia. Although 30 MRI procedures were initially identified in 12 children, several were excluded because the patients either did not receive sedation or were administered sedatives outside the standardized protocol. The limited sample size did not allow for sufficiently robust statistical analysis, increasing the risk of random or marginal findings.

## 5. Conclusions

Standardized procedural sedation using a combination of midazolam and propofol, targeted to achieve an adequate depth of sedation while maintaining airway patency and optimizing imaging conditions, and guided by continuous multimodal monitoring was associated with minimal adverse events and high-quality MRI in pediatric patients with achondroplasia. Although achondroplasia is the most common form of skeletal dysplasia, it remains a rare condition, and clinical data on procedural sedation in this population are limited. Despite the small sample size, our findings support the feasibility and safety of this targeted sedation approach in a carefully monitored setting. The results highlight the importance of specialized anesthetic strategies tailored to the unique anatomical and physiological characteristics of this patient population.

## Figures and Tables

**Table 1:** Patient demographics and number of MRI procedures performed under the standardized sedation protocol.

Patient No.	Sex	Interventions (n)	Age (months)
1	Male	4	18.16–34.10
2	Male	2	0.68–8.21
3	Male	3	12.55–59.76
4	Female	1	4.73
5	Male	2	21.65–55.39
6	Male	2	20.56–70.66
7	Female	1	6.30
8	Female	1	56.34
9	Female	1	27.46
10	Female	2	9.39–15.21
11	Female	2	7.81–46.75
12	Male	1	14.52

Age is presented in months. For patients with multiple procedures, the range of ages at the time of each MRI is provided.

**Table 2:** Sedation parameters and dosing of midazolam and propofol (medians and ranges) under a standardized protocol in 22 MRI procedures in 12 children with achondroplasia.

			Propofol						Midazolam	
Pt No.	S (min)	R (min)	Total (mg)	Total (mg/kg/BW)	IND (mg)	IND2 (mg)	IND (mg/kg/BW)	INF (mg/kg/h)	(mg)	(mg/kg)
1	41.25 25–65	9 7–11	42.5 30–50	4.05 3.37–4.36	12.5 10–20	7.5 5–10	1.55 1.12–2.0	4.04 2.367–5.393	1.125 1–1.5	0.108 0.085–0.136
2	40 30–50	7	26 2–50	3.92 0.49–7.35	11 2–20		1.72 0.49–2.94	5.294	0.75 0.5–1	0.135 0.123–0.147
3	51.67 40–65	7.33 5–9	70 46–104	6.13 5.41–7.14	13.33 10–20	10	1.55 1.12–2.35	5.249 4.588–5.495	2 1.5–3	0.175 0.169–0.179
4	30	9	40	7.13	10	10	3.57	7.13	1	0.178
5	32.5 30–35	9.5 8–11	45 40–50	2.17 4.03–4.30	15 10–20	10	2.29 2.15–2.42	3.457 3.226–3.687	1	0.095 0.081–0.108
6	32.5 30–35	8.5 8–9	45 35–55	7.11 3.23–3.88	15 10–20	7.5 5–10	1.72 1.67–1.76	3.376 2.941–3.810	1.25 1–1.5	0.1 0.088–0.111
7	45	10	15	2.38	10		1.59	1.058	1.5	0.238
8	25	10	80	4.84	30	20	3.03	4.364	1.5	0.091
9	25	10	45	3.84	20		1.71	5.128	1	0.085
10	32.5 30–35	6.5 5–8	34.5 15–54	5.42 2.89–7.94	7.5 5–10	5	1.7 1.47–1.93	7.297 1.652–12.941	0.75 0.5–1	0.122 0.096–0.147
11	40 35–45	11.5 9–14	67.5 35–100	7.24 5.38–9.09	15 10–20	10	2.45 1.82–3.08	6.827 3.956–9.697	1.5 1–2	0.168 0.154–0.182
12	40	9	38	4.81	15		1.9	4.367	1.5	0.190

Data are presented as medians (range) or as absolute numbers. Abbreviations: BW, body weight; IND, induction; INF, infusion; Pt, patient; R, recovery; S, sedation.

**Table 3:** Practical recommendations for procedural sedation during MRI in children with achondroplasia.

Phase	Recommendations
Pre-sedation Assessment	Detailed neurological and airway assessment Polysomnography to evaluate obstructive sleep apnea (OSA) and other sleep-related breathing disorders Plan intravenous access; use near-infrared (NIR) imaging or ultrasound guidance in DIVA patients
Sedation Protocols	Premedication: midazolam (0.1–0.2 mg/kg) Induction: propofol (1–2 mg/kg IV) Maintenance: propofol infusion (3–5 mg/kg/h) Alternative protocols: dexmedetomidine (bolus 0.5–1 µg/kg over 10 min, infusion 0.5–1 µg/kg/h); combination protocols (propofol/dexmedetomidine, ketamine/dexmedetomidine)
Airway Equipment	Difficult airway cart readily available: Video laryngoscope (appropriate pediatric blades) Pediatric fiberoptic bronchoscope Laryngeal mask airways (various pediatric sizes) Oropharyngeal/nasopharyngeal airways Endotracheal tubes (smaller sizes) High-flow nasal oxygen as respiratory support
Airway Safety and Monitoring	Continuous multimodal monitoring (SpO_2_, EtCO_2_, HR, and RR). Neutral cervical spine positioning. Avoid hyperextension. Establish alternative airway management strategies (Plan B). Consider high-flow nasal oxygen if necessary.
Recovery Management	Post-procedural monitoring until modified Aldrete score ≥ 9. Provide discharge instructions emphasizing vigilance for respiratory compromise or airway obstruction.

MRI, magnetic resonance imaging; OSA, obstructive sleep apnea; DIVA, difficult intravenous access; IV, intravenous; HR, heart rate; RR, respiratory rate.

## Data Availability

The datasets used and analyzed during this study are available from the corresponding author upon reasonable request.

## Abbreviations

The following abbreviations are used in this manuscript:

ASA	American Society of Anesthesiologists
DIVA	difficult intravenous access
FMS	foramen magnum stenosis
MRI	magnetic resonance imaging
OSA	obstructive sleep apnea
RR	respiratory rate
UMSS	University of Michigan Sedation Scale
